# Quantitative PCR to Discriminate Between *Pneumocystis* Pneumonia and Colonization in HIV and Non-HIV Immunocompromised Patients

**DOI:** 10.3389/fmicb.2021.729193

**Published:** 2021-10-20

**Authors:** Patsharaporn T. Sarasombath, Jerapas Thongpiya, Monrat Chulanetra, Sirirat Wijit, Pisith Chinabut, Jeerawan Ongrotchanakun, Anupop Jitmuang, Darawan Wanachiwanawin

**Affiliations:** ^1^Department of Parasitology, Faculty of Medicine Siriraj Hospital, Mahidol University, Bangkok, Thailand; ^2^Department of Medicine, Faculty of Medicine Siriraj Hospital, Mahidol University, Bangkok, Thailand

**Keywords:** *Pneumocystis* pneumonia, *Pneumocystis jirovecii*, PCP, colonization, definite PCP, probable PCP, qPCR, *Pneumocystis*

## Abstract

*Pneumocystis* pneumonia (PCP) is an opportunistic infection that commonly occurs in immunocompromised individuals. A definite diagnosis of PCP can be made only when the organism is identified in a respiratory specimen. It remains unclear whether qPCR can differentiate patients with PCP from those with *Pneumocystis jirovecii* colonization. In this study, we retrospectively collected data from HIV and non-HIV patients during 2013–2019. A diagnosis of definite, probable PCP, or PCP excluded was made based on clinical criteria, radiological reports, and three standard laboratory staining methods with blinding to qPCR data. Data from qPCR that was performed to determine the fungal burden (DNA copies/μl) in the BAL specimens of 69 HIV and 286 non-HIV patients were then obtained and reviewed. Receiver Operating Characteristic (ROC) curve analysis was performed to determine the upper and lower cut-off values for PCP diagnosis in HIV and non-HIV groups. In the non-HIV group, the lower cut-off value of 1,480 DNA copies/μl yielded a sensitivity of 100% (95% confidence interval [CI], 91.0–100), specificity of 72.9% (95% CI, 64.0–80.7), a positive predictive value (PPV) of 54.9% (95% CI, 47.6–62.1), and a negative predictive value (NPV) of 100% with Youden index of 0.73 for PCP diagnosis. In this group, the upper cut-off value of 9,655 DNA copies/μl showed the sensitivity of 100% (95% CI, 91.0–100) and specificity of 95.8% (95% CI, 90.4–98.6) with PPV of 88.6% (95% CI, 76.8–94.8) and a NPV of 100% with Youden index of 0.96 for PCP diagnosis. Regarding the HIV group, the lower cut-off value of 1,480 DNA copies/μl showed the sensitivity of 100% (95% CI, 92.5–100%) and specificity of 91.7% (95% CI, 61.5–99.8) with PPV of 97.9% (95% CI, 87.8–99.7) and a NPV of 100% with Youden index of 0.92 for PCP diagnosis. The sensitivity and specificity of the upper cut-off value of 12,718 DNA copies/μl in this group were 97.9% (95%CI, 88.7–100) and 100% (95%CI, 73.5–100), respectively. The values above the upper cut-off point had a PPV of 100% (95% CI, N/A) and a NPV of 92.3% (95% CI, 63.3–98.8) with Youden index of 0.98 for PCP diagnosis in the HIV group.

## Introduction

*Pneumocystis jirovecii* is a fungus that causes life-threatening pneumonia known as *Pneumocystis* pneumonia (PCP) in immunocompromised individuals, including those with HIV and other immunosuppressive conditions (Kovacs and Masur, 2009; Roux et al., 2014). A few decades ago, the disease mainly affected people with HIV, and it used to be one of the most common opportunistic infections (Morris et al., 2004; Roux et al., 2014). Importantly, the incidence of PCP in HIV has significantly declined since the beginning of the global use of highly active antiretroviral therapy (HAART; Morris et al., 2004; Dunbar et al., 2020). However, the increasing use of immunosuppressive medication, chemotherapeutic agents, and advancements in organ transplantation has significantly increased the risks of PCP among non-HIV immunosuppressive individuals (Morris et al., 2004; Dunbar et al., 2020).

The clinical presentations of PCP in HIV-infected patients include the typical triad of dyspnea, fever, and cough; however, the symptoms in non-HIV patients are often acute, progressive with atypical presentations, and high mortality rates (Thomas and Limper, 2004; Calderón et al., 2010; Roux et al., 2014). Moreover, non-HIV patients usually present with a lower fungal burden in the respiratory tract compared to the fungal burden in HIV-infected individuals (Limper et al., 1989; Calderón et al., 2010). Thus, diagnosis of PCP in non-HIV patients is often challenging.

The diagnosis of PCP relies mainly on clinical presentations, patient history, and laboratory results. Identification of the organism in a respiratory specimen by microscopic-based staining methods, such as Giemsa, Gomori methenamine silver (GMS), calcofluor white, toluidine blue, and/or immunofluorescence antibody assay (IFA), is required for definite diagnosis of PCP (Calderón et al., 2010; Tomás and Matos, 2018). A standard laboratory-based diagnosis may give false-negative results especially in patients with a low organism burden. Due to recent advances in molecular diagnosis, various techniques, including quantitative PCR (qPCR), are now widely used for the detection of *P. jirovecii* in clinical specimens due to their high sensitivity and specificity (Calderón et al., 2010; Tomás and Matos, 2018). Thus, qPCR is a useful tool for PCP diagnosis especially in non-HIV immunocompromised patients with a low organism burden and atypical clinical presentations. Although a negative PCR result in respiratory specimens supports the exclusion of PCP diagnosis, a positive result alone cannot be used to confirm the diagnosis of PCP (Tomás and Matos, 2018). A positive PCR result may reflect colonization of *P. jirovecii* in the respiratory tract and may not correlate with patient clinical manifestations (Morris and Norris, 2012; Tomás and Matos, 2018). One of the drawbacks of qPCR is its inability to distinguish active PCP from colonization (Morris and Norris, 2012). An increase in the organism burden in respiratory specimens is often correlated with a higher probability of a patient having active PCP (Calderón et al., 2010; Tomás and Matos, 2018). Previous studies have proposed upper and lower qPCR cut-off levels with high sensitivity and specificity for distinguishing active PCP from colonization in HIV and non-HIV patients (Linssen et al., 2006; Huggett et al., 2008; Alanio et al., 2011; Botterel et al., 2012; Mühlethaler et al., 2012; Maillet et al., 2014; Fauchier et al., 2016; Bossart et al., 2020; Perret et al., 2020). In the present study, we set forth to determine if our in-house qPCR targeting the mitochondrial large-subunit ribosomal RNA (mtLSU-rRNA) can differentiate PCP from colonization based on the fungal burden in bronchoalveolar lavage (BAL) specimens from HIV and non-HIV patients.

## Materials and Methods

### Study Population, Data Collection, and Clinical Samples

The retrospective study was conducted at the Faculty of Medicine Siriraj Hospital, Mahidol University, Bangkok, Thailand, and the study protocol was approved by the Siriraj Institutional Review Board of research involving human subjects (SIRB; COA no. Si163/2020). The clinical data of patients whose BAL samples were sent for diagnosis from January 2013 to December 2019 were included. Approximately, 500 patient data were reviewed and only 355 patients whose BAL and clinical data can be retrieved were included in the study. Patient information, including age, gender, underlying diseases, clinical symptoms, radiological reports, PCP prophylaxis, medical treatments, laboratory data, and treatment outcomes were collected from our center’s electronic database.

### Definitions for Diagnosis

Two physicians independently reviewed the patient records and classified them into three groups: (1) definite PCP, (2) probable PCP, and (3) PCP excluded based on patients and laboratory data from the electronic database. Both physicians were blinded to the qPCR results during this phase of the study. The diagnostic criteria used to classify patients were listed in Table 1. After the patients were classified into the three aforementioned groups, the qPCR data were obtained. Patients in the PCP excluded group were later recategorized into the colonization or PCP negative groups based on the qPCR results. The negative group was defined as cases that had a fungal burden of ≤100 DNA copies/μl. The colonization group did not meet the criteria for PCP diagnosis, but *P. jirovecii* was identified in BAL samples by qPCR with a fungal burden >100 DNA copies/μl.

**Table 1:** Criteria used for diagnosis definition. Mandatory criteria must be met for each diagnosis.

**Definite PCP**	
-	Having at least three out of four clinical signs of pneumonia including fever, cough, dyspnea/tachypnea, and hypoxia
-	^*^Chest imaging was compatible with acute pneumonia.
-	^**^Identification of *P. jirovecii* in BAL specimens by ≥ 2 out of three microscopic-based methods including GMS stain, Giemsa stain, and IFA
**Probable PCP**	
-	Having at least three out of four clinical signs of progressive pneumonia including fever, cough, dyspnea/tachypnea, and hypoxia
-	Either a chest imaging compatible with PCP (diffuse bilateral reticulonodular or granular infiltration), but with an absence of microscopic identification of *P. jirovecii* in BAL specimens or ^**^identification of *P. jirovecii* in BAL specimens by only one microscopic-based method including GMS stain, Giemsa stain, and IFA
**PCP excluded (colonization or negative)**	
-	No sign of pneumonia or having pneumonia from other pathogens besides PCP
-	A chest imaging was normal or incompatible with PCP.
-	Absence of microscopic identification of *P. jirovecii* in BAL specimens by GMS stain, Giemsa stain, and IFA
-	An alternative diagnosis was made.

*PCP, Pneumocystis pneumonia; P. jirovecii, Pneumocystis jirovecii; BAL, bronchoalveolar lavage; GMS stain, Gomori methenamine silver stain; IFA, immunofluorescence antibody assay; and qPCR, quantitative polymerase chain reaction*.

* *Chest imaging in the definite PCP included bilateral reticulonodular infiltration (n=56, 83.6%), localized reticulonodular infiltration (n=5, 7.4%), bilateral interstitial infiltration and alveolar infiltration (n=2, 3%), bilateral alveolar infiltration (n=2, 3%), localized alveolar infiltration (n=1, 1.5%), and localized interstitial infiltration with multiple thick wall cavities (aspergilloma) and pleural effusion (n=1, 1.5%)*.

** *Presence of cystic form in BAL by GMS stain, presence of cystic and tropic forms in BAL by Giemsa stain or IFA*.

### Sample Collection, Staining, and DNA Extraction

A total of 355 BAL samples from patients whose clinical data were available in the database were subjected to qPCR, IFA, Giemsa staining, and GMS staining. The BAL specimens were stored in −20°C before analysis. Approximately, 3–5ml of BAL specimen of each sample was briefly mixed and then centrifuged at 2,000g for 10min at 4°C. The supernatant was removed, and approximately, 1ml of the BAL precipitate remained. Two hundred microliters of BAL precipitate were subjected to DNA purification. For qPCR, the precipitates were subjected to DNA extraction by the QIAmp DNA Mini Kit (Qiagen, Hilden, Germany) following the manufacturer’s protocol for isolating DNA from body fluids. DNA purity and concentration of each sample were measured by NanoDrop 2000 UV-Vis Spectrophotometer (Thermo Fisher Scientific, Waltham, MA, United States). Twenty-five microliters of the precipitate were used for IFA, Giemsa, and GMS staining. The Giemsa and GMS staining methods were performed following the protocols as previously described (Garcia, 2007). For IFA, mouse monoclonal antibody (3F6; Dako, Glostrup, Denmark) was used to stain cystic and tropic forms of *P. jirovecii* as previously described (Linder et al., 1986; Elvin et al., 1988). The conventional staining methods were performed and independently analyzed by three microscopists, and all microscopists had to be in agreement. The persons who performed these analyses were all blinded to qPCR data.

### Quantitative PCR for *P. jirovecii* Detection

Quantitative PCR targeting *P. jirovecii* mitochondrial large-subunit ribosomal RNA (mtLSU-rRNA), which yields the amplicon size of 168 base pairs (bp), was performed as previously described (Sarasombath et al., 2020). The forward and reverse primers used for mtLSU-rRNA were 5′-GGCATAACTCATGCTTAACAGT-3′ and 5′-GGCAAACTTCGTGGTCAATAC-3′, respectively. Quantitative PCR for human glyceraldehyde-3-phosphate dehydrogenase (GAPDH), a housekeeping gene, was also performed to assure the DNA sample quality. The forward and reverse primers used for human GAPDH were 5′-AGGTCATCCCTGAGCTGAA-3′and 5′-CTGCTTCACCACCTTCTTGAT-3′. The GAPDH amplion size is 137bp. Only the samples that the human GAPDH was amplified were further proceeded for *P. jirovecii* qPCR. The plasmids of *P. jirovecii* mtLSU-rRNA were used as standards for DNA copy number quantification as previously described (Sarasombath et al., 2020). In brief, eight standards ranging from 1×10^8^ to 1×10^1^ copies/μl of the plasmid, a water control, a negative control, and a positive control sample were included in each run. All the samples were run in triplicate. Thermocycling and fluorescence detection were performed with a LightCycler^®^ 480 Instrument II (Roche Applied Science, United States). Reactions were set up using LightCycler^®^ 480 SYBR Green I Master in a total volume of 10μl that contained 1μl of DNA, 1pmol of each primer, and 2μl of H_2_O in 5μl of LightCycler^®^ 480 SYBR Green I Master (2x). LightCycler^®^ Uracil-DNA glycosylase was added to the master mix at the final concentration of 0.5U per 20μl reaction to prevent carryover contamination in PCR. The cycling conditions were as follows: Uracil-DNA glycosylase reaction at 40°C for 10min; initial denaturation at 95°C for 10min; 45cycles of 95°C for 10s, 55°C for 20s, and 72°C for 20s; and the melting analysis ranged from 95 to 65°C. A melt curve analysis was performed in each run at the end of PCR cycles to confirm the presence of a single specific amplicon only for mtLSU-rRNA and GAPDH. The quantity of *P. jirovecii* in samples was determined according to a standard curve. The results are expressed as the numbers of *P. jirovecii* DNA copies/μl.

### Statistical Analysis

Data were analyzed using SPSS Statistics version 18 (SPSS, Inc., Chicago, IL, United States). Patient demographic and clinical data were analyzed using descriptive statistics. Continuous variables were compared using Kruskal-Wallis one-way ANOVA due to the non-symmetrical distribution of the data and those results are reported as median and interquartile range. GraphPad Prism 7.00 (GraphPad Software, Inc., San Diego, CA, United States) was used to generate scatter plots and calculating a Receiver Operating Characteristic (ROC) curve analysis, sensitivity, specificity, positive predictive value (PPV), negative predictive value, and likelihood ratio. Youden index was calculated using MedCalc Statistical Software version 19.2.6 (MedCalc Software Ltd., Ostend, Belgium; https://www.medcalc.org; 2020). ROC analysis of DNA copies/μl was used to define cut-off values to distinguish the definite and probable PCP from the colonization group. The cut-off values were defined based on the criteria that no probable PCP was diagnosed in the patients with a fungal burden less than the lower cut-off values; while, no definite PCP was diagnosed in the patients with a fungal burden lower than the upper cut-off values. Data in qPCR negative groups were not included in the scatter plots and ROC analysis. A value of *p*≤0.05 (two tailed) was considered to be statistically significant.

## Results

### Overall Clinical Data and Classification of the Study Groups

Data and samples from 355 patients were included in this study. The diagnostic criteria used for the classification of *Pneumocystis* infection-based groups (definite PCP, probable PCP, and PCP excluded) were listed in Table 1. The overall demographic data and clinical characteristics of the patients included in this study were demonstrated in Table 2. We found that 86 (24.2%) of the study patients were diagnosed with PCP including those of 67 (18.9%) definite and 19 (5.3%) probable PCP, respectively (Table 2). In the definite PCP group, BAL samples of 63 (94%) and 4 (6%) patients were identified by all or two microscopic-based methods (GMS, Giemsa, or IFA). In the probable PCP group, BAL samples from four (21.1%) patients were positive by one microscopic-based method, and there were 15 (78.9%) patients for whom *P. jirovecii* was not detected in BAL by microscopic-based methods, but chest imaging and clinical characteristics were compatible with PCP. Thirteen (86.7%) of 15 patients in that group received anti-*Pneumocystis* medications with subsequent clinical improvement. Patients defined as definite or probable PCP had at least one immunological disorder. In the definite PCP group, 42 (62.7%) patients had HIV, 22 (32.8%) were non-HIV with other immunological disorders, and three (4.5%) patients had more than one immunosuppressive condition. In the probable PCP group, two (10.5%) patients had HIV, and 17 (89.5%) were non-HIV with other immunocompromised conditions. The colonization and negative groups comprised the following; nine (6.9%) and eight (5.8%) patients with HIV; 95 (73.1%) and 83 (59.7%) patients with other immunodeficiency conditions; four (3.1%) and four (2.9%) patients with more than one immunosuppressive conditions; and 22 (16.8%) and 44 (31.7%) patients with no known immunological disorders, all, respectively. Only eight (11.9%) patients in the definite PCP group received PCP prophylaxis while none of the probable PCP patients received the prophylaxis. Twenty-eight (21.5%) and 25 (18%) patients in the colonization and negative groups received PCP prophylaxis, respectively. The main reason why the majority of PCP patients did not receive PCP prophylaxis was either medication discontinuation or first diagnosis of HIV.

**Table 2:** Patient demographic data and clinical characteristics compared among the *Pneumocystis* infection-based classification (*N*=355).

Characteristics	Definite PCP (*n*=67)	Probable PCP (*n*=19)	Colonization (*n*=130)	Negative (*n*=139)
No. of male patients (%)	48(71.6)	11(57.9)	65(50.0)	70(50.4)
Median age in years (IQR)	40(30.0–49.0)	57(44.0–69.0)	55(14.0–64.0)	50(29.0–66.0)
No. of patients with HIV (%)	42(62.7)	2(10.5)	9(6.9)	8(5.8)
Median numbers of CD4 count in mm^3^ (IQR)	30(10.0–41.0)	N/A	86(29.0–150.0)	81(6.5–364.0)
No. of patients with the following underlying immunodeficiency conditions besides HIV (%)	22(32.8)	17(89.5)	95(73.1)	83(59.7)
Hematologic malignancy	11(16.4)	2(10.5)	32(24.6)	24 (17.3)
Solid organ transplant	6(9.0)	1(5.3)	9(6.9)	10(7.2)
Autoimmune disorder	5(7.5)	9(47.4)	40(30.8)	37(26.6)
Solid organ malignancy	0(0)	2(10.5)	7(5.4)	6(4.3)
Congenital immune disorders	0(0)	0(0)	2(1.5)	2 (1.4)
Others	0(0)	3(15.8)	5(3.8)	4(2.9)
No of patients with more than one immunosuppressive conditions (%)	3(4.5)^α^	0(0)	4(3.1)^β^	4(2.9)^γ^
No. of patients with no known immunological disorders^*^	0(0)	0(0)	22(16.9)	44(31.6)
No. of patients receiving PCP prophylaxis (%)	8(11.9)	0(0)	28(21.5)	25(18.0)
No. of patients with the following treatment (%)				
Anti-Pneumocystis medications^¶^	13(19.4)	10(52.6)	39(30.0)	32(23.0)
Anti-Pneumocystis + corticosteroids	47(70.1)	6(31.6)	5(3.8)	8(5.8)
Other antibiotic treatment	54(80.6)	18(94.7)	99(76.2)	28(20.1)
Mechanical ventilation	16(23.9)	6(31.6)	40(30.8)	54(38.8)
Fungal burden (DNA copies/μl) as assessed by qPCR (IQR)	4.6×10^5^(1.5×10^5^–1.0×10^6^)	5.3×104(3.0×10^4^–1.0×10^5^)	560(280–1,510)	UD
Fungal burden in patients with HIV^*^	4.8×10^5^(1.9×10^5^–1.4×10^6^)	2.3×10^4^(NA)	436(365–934)	UD
Fungal burden in patients with other non-HIV immune deficiency conditions	4.2×10^5^(1.0×10^5^–7.9×10^5^)	5.7×10^4^(1.4×10^4^–1.1×10^5^)	585(268.8–1,762.5)	UD

* *Patients with more than one immunosuppressive condition who were co-infected with HIV were included in the HIV for dot-plot and ROC curve analysis*.

¶ *Anti-pneumocystis medications included first line: co-trimoxazole and second line: primaquine and clindamycin or pentamidine*.

α *One patient with HIV and DLCBL, one patient with HIV and cervical carcinoma, and one patient with HIV and CNS lymphoma*.

β *One patient with SLE and DLCBL, one patient with HIV and lung adenocarcinoma, and two patients with HIV and Evan’s syndrome*.

γ *One patient with SLE and DLCBL, one patient with RA and MM, and two patients with HIV and DLCBL (N/A, not available; UD, undetectable; HIV, human immunodeficiency virus; CNS, central nervous system; DLCBL, diffuse large B-cell lymphoma; SLE, systemic lupus erythematosus; RA, rheumatoid arthritis; and MM, multiple myeloma)*.

The clinical characteristics of the patients in each PCP group were further subcategorized into HIV and non-HIV subgroups. Patients with HIV and one or more other immunosuppressive conditions were included in the HIV group (Table 2). Three, three, and two patients in the definite, colonization, and negative groups who had one or more immunosuppressive conditions in addition to HIV were included in the HIV group (Footnote, Table 2 and Supplementary Table S1). The demographic data of the non-HIV immunocompromised patients were listed in Supplementary Table S1, with hematologic malignancy being the most common immunosuppressive condition in the non-HIV group. Twenty-two (16.8%) and 44 (31.7%) patients in the colonization and negative groups had no known immunological disorders (Table 2).

### Comparison of Fungal Burdens Among *Pneumocystis* Infection-Based Groups in HIV and Non-HIV Patients

**Figure 1:**
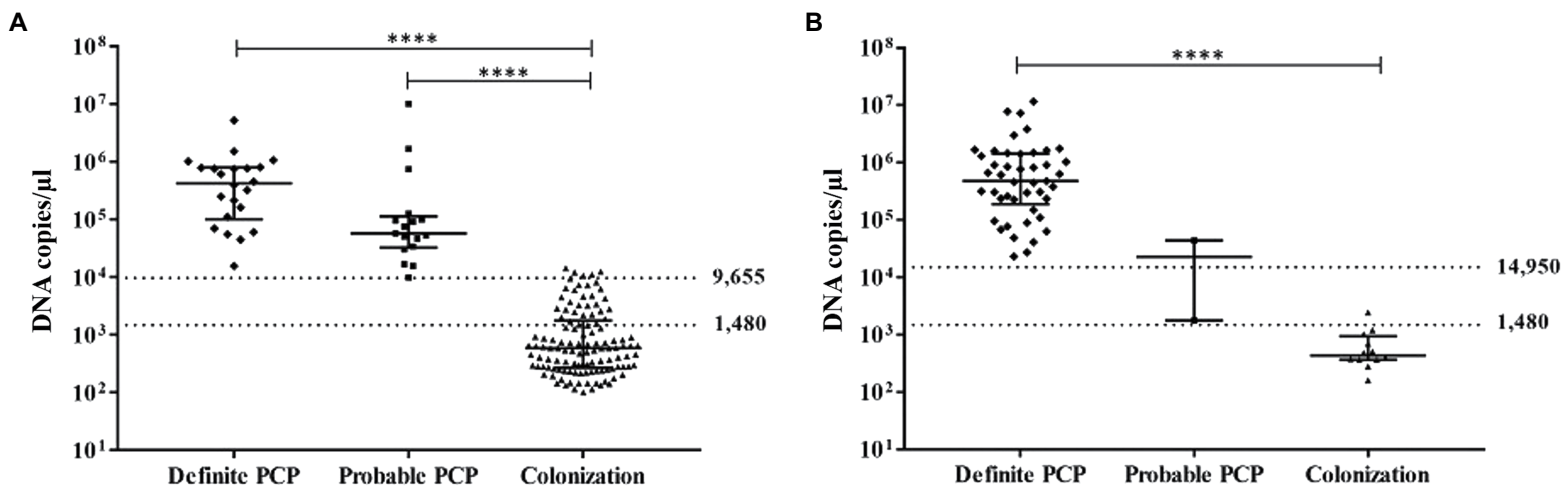
Scatter plot showing *P. jirovecii* mtLSU-rRNA DNA copies/μl in bronchoalveolar lavage (BAL) samples among definite PCP, probable PCP, and colonization in non-HIV **(A)** and HIV **(B)** groups. Each symbol represents the number of DNA recovered from each patient. The horizontal bars represent the median values with IQR. The dot lines showed the upper and lower cut-off values in HIV and non-HIV groups. Analysis was performed using the non-parametric one-way ANOVA (Kruskal-Wallis) test. ^****^value of p <0.0001.

Fungal burdens of *P. jirovecii* in BAL of the study groups as assessed by qPCR were significantly higher in the definite PCP [4.6×10^5^, interquartile range (IQR) 1.5×10^5^–1×10^6^] than in the probable PCP (5.3×10^4^, IQR 3×10^4^–1×10^5^), and the colonization groups (560, IQR 280-1,510; Table 2; Figures 1A,B). The overall fungal burdens were significantly higher among definite PCP and probable PCP as compared to colonization and negative groups (*p*<0.0001). Fungal burden data in each study group in non-HIV and HIV individuals were shown in Table 2 and Figures 1A,B. The fungal burdens in the definite and probable PCP in HIV-infected patients were slightly higher than the numbers in the non-HIV group with no statistical significance (Table 2; Figures 1A,B).

**Figure 2:**
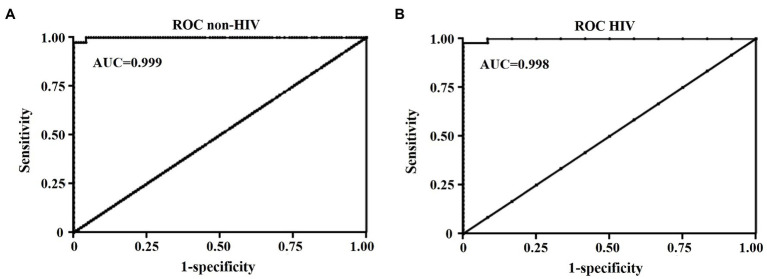
Receiver Operating Characteristic (ROC) curve analysis of DNA copies/μl of BAL samples for the diagnosis of PCP in non-HIV **(A)** and HIV **(B)** groups. AUC, an area under the curve.

ROC curve analysis was performed to define cut-off values to discriminate the definite and probable PCP groups from the colonization group in HIV and non-HIV. The qPCR data of the negative group were not included in the analysis to avoid data bias. ROC curve analysis gave an area under the curve of 0.999 for the non-HIV group (Figure 2A). The lower and upper cut-off values in non-HIV and HIV groups were proposed to differentiate the diagnosis of PCP (definite/probable PCP) and colonization in each group. The cut-off values were defined based on the criteria that no probable PCP was diagnosed in the patients with a fungal burden less than the lower cut-off values; while, no definite PCP was diagnosed in the patients with a fungal burden lower than the upper cut-off values. In the non-HIV group, the lower cut-off value of 1,480 DNA copies/μl yielded the sensitivity and specificity of 100% [95% confidence interval (CI), 91.0–100] and 72.9% (95% CI, 64.0–80.7), respectively. The values above this cut-off point had a PPV of 54.9% (95% CI, 47.6–62.1) with a positive likelihood ratio (LR) of 3.69 (95% CI, N/A), and a negative predictive value (NPV) of 100% for the diagnosis of PCP in the non-HIV group. This cut-off value yields the Youden index of 0.73. With the upper cut-off value of 9,655 DNA copies/μl, the sensitivity and specificity were 100% (95% CI, 91.0–100%) and 95.8% (95% CI, 90.4–98.6), respectively. The values above this cut-off point had a PPV of 88.6% (95% CI, 76.8–94.8) with a positive LR of 23.6 (95% CI, 10.0–55.7) and a NPV of 100% for the diagnosis of PCP in the non-HIV group. This cut-off value gave the Youden index of 0.96. The colonization group was defined by the absence of PCP-related clinical and radiological features, and the absence of the organism in BAL by the three standard microscopic-based methods (Table 1), but showed the presence of the organism by qPCR. A fungal burden ranged between 1,480 and 9,655 DNA copies/μl in the non-HIV patients, suggested possible colonization.

Regarding the HIV group, ROC curve analysis gave an area under the curve of 0.998 (Figure 2B). The lower cut-off value of 1,480 DNA copies/μl yielded the sensitivity and specificity of 100% (95% CI, 92.5–100%) and 91.7% (95% CI, 61.5–99.8), respectively. The values above this lower cut-off point have a PPV of 97.9% (95% CI, 87.8–99.7) with a positive LR of 12 (95% CI, 1.8–78.4), and a NPV of 100% for the diagnosis of PCP in HIV group with the Youden index of 0.92. The sensitivity and specificity of the upper cut-off value of 12,718 DNA copies/μl were 97.9% (95%, CI 88.7–100) and 100% (95% CI, 73.5–100), respectively. The values above the upper cut-off point had a PPV of 100% (95% CI, N/A) with a negative LR of 0.02 (95% CI, 0–0.15) and a NPV of 92.3% (95% CI, 63.3–98.8) for the diagnosis of PCP in the HIV group with the Youden index of 0.98. A fungal burden ranged between 1,480 and 12,718 DNA copies/μl in HIV patients, suggested possible colonization in the HIV group. Therefore, the fungal burdens in BAL fluid can be used to distinguish *P. jirovecii* pneumonia from colonization in both HIV and non-HIV groups.

## Discussion

It is still being investigated and debated whether the assessment of fungal burden by qPCR in patient respiratory specimens can be used to distinguish active PCP from colonization. Several studies have proposed qPCR for differentiation of patients with active PCP and colonization with promising results (Alanio et al., 2011; Botterel et al., 2012; Mühlethaler et al., 2012; Maillet et al., 2014; Fauchier et al., 2016; Bossart et al., 2020; Perret et al., 2020). Despite the similarity of using the qPCR technique for estimation of fungal burden, there are some notable differences among those previously reported studies including the study designs, the target genes: single copy genes [heat shock protein 70 (Huggett et al., 2008) and dihydropteroate synthase (Linssen et al., 2006)] or multiple copy genes [mitochondrial large-subunit rRNA (Alanio et al., 2011; Botterel et al., 2012; Robert-Gangneux et al., 2014; Fauchier et al., 2016), mitochondria 26S rRNA (Perret et al., 2020), and major surface glycoprotein (Linssen et al., 2006; Maillet et al., 2014)]. Moreover, the clinical criteria used for PCP diagnosis, the types of respiratory specimens (BAL, sputum, and oral wash), the demographic data of the study subject, and the fungal burden unit (DNA copies per ml, cycle threshold, and trophic form equivalents per ml) and the cut-off values were distinct among the studies. These differences make it difficult to directly compare the data and findings among those studies. The majority of those studies demonstrated the ability of qPCR to differentiate between active PCP and colonization with high sensitivity and specificity (Fauchier et al., 2016; Perret et al., 2020). Various cut-off points, including either a single cut-off or upper or both upper and lower cut-off values, have been purposed to determine the diagnosis of PCP. Alternatively, some studies proposed undetermined values between upper and lower cut-off values which the diagnosis of PCP or colonization is uncertain, and additional data are needed for diagnosis (Alanio et al., 2011; Mühlethaler et al., 2012; Robert-Gangneux et al., 2014). Generally, respiratory specimens from HIV-infected patients have a markedly higher fungal density than those obtained from non-HIV patients (Robert-Gangneux et al., 2014). In patients with non-HIV, strong data suggest the use of qPCR for diagnosis of PCP because of low fungal burden and atypical clinical presentations of PCP in this group (Botterel et al., 2012; Maillet et al., 2014; Robert-Gangneux et al., 2014; Fauchier et al., 2016). The role of qPCR for the diagnosis of PCP in HIV-infected individuals is still debatable. Previous studies suggested defining a cut-off value in HIV may not be essential, and qPCR should not be used as an initial laboratory investigation in this group (Alanio et al., 2011).

In this study, we determined to test whether our in-house qPCR method can discriminate PCP from colonization in HIV and non-HIV immunocompromised individuals. We chose a multiple copy gene (mtLSU-rRNA) to increase the sensitivity of qPCR. In this study, a diagnosis of definite PCP or probable PCP was made with stringent clinical and laboratory criteria, and while blinded to the qPCR results. Three conventional microscopic-based methods including Giemsa stain, GMS stain, and IFA were performed in this study to diminish the possibility of false-positive or false-negative results. The internal control of a housekeeping gene to assure DNA sample quality was also included in each qPCR reaction. An Uracil-DNA glycosylase treatment was also performed in each run to minimize the possibility of DNA carryover among samples.

However, this study has some limitations. This was a single-center retrospective study; thus, clinical data, laboratory data, and diagnosis were collected retrospectively. As such, the technique used, the location from where the specimen was collected, and the volume of BAL specimen collection could vary from sample to sample. Moreover, the fungal burden in BAL may not directly reflect the numbers of cystic or trophic forms and the physiological state of the organism (Valero et al., 2016). The information relating to whether different stages of the organism reflect the disease phenotype is unknown (Valero et al., 2016). Additional tests, such as serum (1–3)-beta-D-glucan (BG) and lactate dehydrogenase (LDH) and Krebs von den Lungen-6 antigen (KL-6) tests, that have been previously shown to aid the diagnosis of PCP were not be performed in our study (Nakamura et al., 2009; Damiani et al., 2013; Esteves et al., 2015). Unfortunately, BG and KL-6 tests were not yet available at our institute, and serum LDH was not performed in most cases.

Quantitative PCR significantly increases the sensitivity of *P. jirovecii* detection in BAL as compared to the conventional staining methods. This is especially true in the patients in the non-HIV group that have low fungal burdens and atypical clinical presentations. By way of example, the organism was not detected by the conventional staining methods in 15 of 17 (88.2%) BAL samples from probable PCP in the non-HIV group. In our study, we found a direct correlation between the fungal burden and diagnosis of PCP. Moreover, we proposed that our in-house qPCR can be used to exclude the diagnosis of PCP since none of the BAL samples of the patients in the negative PCP group had positive qPCR results. The median fungal burden among patients diagnosed with PCP in the HIV-infected group was higher than the median fungal burden among patients diagnosed with PCP in the non-HIV group. This finding is consistent with those from several studies which found that PCP develops with a low fungal burden in non-HIV immunocompromised individuals. Interestingly, we also found that only a few patients with HIV had fungal burdens in BAL samples which fell in the gray zone. This is similar to the findings observed in another study which showed that HIV-infected patients were more likely to be diagnosed with PCP by direct examination as compared to non-HIV immunocompromised patients (Robert-Gangneux et al., 2014). This may be due to higher amounts of the organism in the respiratory tract of HIV-infected patients as compared to other immunocompromised individuals, which may facilitate earlier diagnosis of PCP in HIV patients due to their having typical clinical presentations of PCP. Moreover, BAL may not be performed in HIV patients with no respiratory symptoms; thus, the colonization in this group may be underestimated. Patients with hematological malignancy were the majority of the patients in the non-HIV group. We also found that the patients in this group had a bleeding tendency which increased the risk of bleeding during the bronchoscopic procedure. We noticed that identification of *P. jirovecii* by the standard staining methods in the BAL samples that contained blood was challenging and the organism can be easily overlooked.

We purposed the two cut-off values (an upper and lower cut-off value) for PCP diagnosis in HIV and non-HIV immunocompromised individuals. A fungal burden above the upper cut-off values is strongly associated with PCP diagnosis in both HIV and non-HIV groups. A fungal burden that falls into the gray zone should be augmented with clinical, radiological, and other laboratory data to arrive at a diagnosis and a treatment decision. Additional laboratory investigations including serum LDH, KL-6 test, and BG may suggest the use of anti-PCP treatment or prophylaxis (Nakamura et al., 2009; Damiani et al., 2013; Esteves et al., 2015).

We concluded that our in-house qPCR assay proposed in this study has high sensitivity, PPV, and NPV for the diagnosis of PCP using the proposed upper cut-off values in both HIV and non-HIV immunocompromised individuals. This test will be particularly useful in patients with clinically suspected PCP with negative conventional laboratory stainings and low fungal burden. Additional tests, patient clinical data, and radiological findings must be considered to support or exclude the diagnosis of PCP in patients with a fungal burden that fell between the upper and lower cut-off values. Validation of the cut-off points proposed in this study for PCP diagnosis in a prospective study and other types of respiratory specimens is still ongoing. This information is crucial for PCP diagnosis and therapeutic decision making, especially in immunocompromised patients.

## Data Availability Statement

The original contributions presented in the study are included in the article/Supplementary Material, further inquiries can be directed to the corresponding authors.

## Ethics Statement

The studies involving human participants were reviewed and approved by The Siriraj Institutional Review Board of research involving human subjects (SIRB), Faculty of Medicine Siriraj Hospital, Mahidol University, Thailand (COA no. Si163/2020). Written informed consent for participation was not required for this study in accordance with the national legislation and the institutional requirements.

## Author Contributions

JT, AJ, and PS reviewed and retrospectively collected clinical data. SW, PC, JO, and MC collected the laboratory data and performed the laboratory diagnoses including GMS stain, Giemsa stain, IFA, and qPCR. PS and DW conceived, designed, and performed the statistical analysis as well as wrote and finalized the manuscript. All authors have read and reviewed the manuscript before submission.

## Funding

This study was funded by a grant from the Routine to Research Unit (R2R), Faculty of Medicine Siriraj Hospital, Mahidol University (grant number RO16335026) awarded to PS and DW. PS, MC, AJ, and DW also received Chalermphrakiat grant from the Faculty of Medicine Siriraj Hospital, Mahidol University.

## Conflict of Interest

The authors declare that the research was conducted in the absence of any commercial or financial relationships that could be construed as a potential conflict of interest.

## Publisher’s Note

Publisher’s Note: All claims expressed in this article are solely those of the authors and do not necessarily represent those of their affiliated organizations, or those of the publisher, the editors and the reviewers. Any product that may be evaluated in this article, or claim that may be made by its manufacturer, is not guaranteed or endorsed by the publisher.
