# Supplementary material for: Quantitative PCR to Discriminate Between Pneumocystis Pneumonia and Colonization in HIV and Non-HIV Immunocompromised Patients
Source: Front Microbiol. 2021 Oct 20;12:729193. doi: 10.3389/fmicb.2021.729193 (PMC8564139; doi:10.3389/fmicb.2021.729193)
Supplement: Supplementary file 1 [file Data_Sheet_1.docx]

**Supplementary Table 1:** Demographic distribution and clinical characteristics of non-HIV immunocompromised study patients based on *Pneumocystis* infection-based classification. Patients with HIV co-infection were excluded.

| **Characteristics, n (%)** | **Definite PCP**  **(n = 22)** | **Probable PCP**  **(n = 17)** | **Colonization**  **(n = 96)** | **Negative**  **(n = 85)** |
| --- | --- | --- | --- | --- |
| **Hematologic malignancy** | **11 (50.0)** | **2 (11.8)** | **32 (33.3)** | **24 (28.2)** |
| Lymphoproliferative disorders |  |  |  |  |
| ALL | 2 (18.2) | 0 (0) | 3 (9.3) | 3 (12.5) |
| NHK lymphoma |  |  |  |  |
| DLCBL | 6 (54.5) | 1 (50.0) | 10 (31.3) | 4 (16.7) |
| MM | 0 (0) | 1 (50.0) | 2 (6.3) | 1 (4.2) |
| Others^α^ | 0 (0) | 0 (0) | 4 (12.5) | 6 (25.0) |
| HK lymphoma | 1 (9.1) | 0 (0) | 4 (12.5) | 2 (8.2) |
| CLL | 0 (0) | 0 (0) | 3 (9.3) | 0 (0) |
| Myeloproliferative disorders |  |  |  |  |
| AML | 2 (18.2) | 0 (0) | 6 (18.8) | 4 (16.7) |
| CML | 0 (0) | 0 (0) | 0 (0) | 4 (16.7) |
| **Solid organ transplant** | **6 (27.3)** | **1 (5.9)** | **9 (9.4)** | **10 (11.8)** |
| Kidney | 5 (83.3) | 0 (0) | 6 (66.7) | 7 (70.0) |
| Liver | 1 (16.7) | 1 (100.0) | 2 (22.2) | 2 (20.0) |
| Lung | 0 (0) | 0 (0) | 1 (11.1) | 0 (0) |
| Heart and kidney | 0 (0) | 0 (0) | 0 (0) | 1 (10.0) |
| **Solid organ malignancy** | **0 (0)** | **2 (11.8)** | **7 (7.3)** | **6 (7.0)** |
| Breast | 0 (0) | 1 (50.0) | 3 (42.9) | 2 (33.3) |
| Lung | 0 (0) | 0 (0) | 3 (42.9) | 0 (0) |
| Brain | 0 (0) | 0 (0) | 1 (14.3) | 1 (16.7) |
| Others^β^ | 0 (0) | 1 (50.0) | 0 (0) | 3 (50.0) |
| **Autoimmune disorder** | **5 (22.7)** | **9 (52.9)** | **40 (41.7)** | **37 (43.5)** |
| SLE | 1 (20.0) | 2 (22.2) | 21 (52.5) | 18 (48.6) |
| RA | 0 | 1 (11.1) | 6 (15.0) | 3 (8.1) |
| Systemic sclerosis | 0 (0) | 0 (0) | 3 (7.5) | 5 (13.5) |
| Others^γ^ | 4 (80.0) | 6 (66.7) | 10 (25.0) | 11 (29.8) |
| **Congenital immune disorders** | **0 (0)** | **0 (0)** | **2 (2.1)** | **2 (2.4)** |
| SCID | 0 (0) | 0 (0) | 1 (50.0) | 0 (0) |
| Hyper IgM syndrome | 0 (0) | 0 (0) | 1 (50.0) | 0 (0) |
| Primary hypogammaglobulinemia | 0 (0) | 0 (0) | 0 (0) | 2 (100) |
| **Other immunosuppressive conditions^δ^** | **0 (0)** | **3 (17.6)** | **5 (5.2)** | **4 (4.7)** |
| **No of patients with more than 1 immunosuppressive condition except HIV (%)** | **0 (0)** | **0 (0)** | **1 (1.0)** | **2 (2.4)** |
| Autoimmune disorders with hematological malignancy | 0 (0) | 0 (0) | 1 (100) | 2 (100) |

DLCBL, Diffuse large B-cell lymphoma; AML, acute myeloid leukemia; ALL, acute lymphocytic leukemia; NHK, Non-Hodgkin’s lymphoma; HL, Hodgkin’s lymphoma; MM, multiple myeloma; CML, chronic myelogenous leukemia; CLL, chronic lymphoid leukemia; SLE, systemic lupus erythematosus, Ig, immunoglobulin

^α^Other non-Hodgkin’s lymphoma included B-cell lymphoma (marginal zone B-cell, mantle cell, follicular B-cell, small lymphocytic), and T-cell lymphoma (adult T-cell leukemia, cutaneous T cell).

^β^Other solid organ malignancy included colon, corpus luteum, germ cell, and neuroendrocrine tumor.

^γ^Other autoimmune diseases included Wegener's granulomatosis, Pauci-immune glomerulonephritis, dermatomyositis, polymyositis, limited scleroderma, antiphospholipid syndrome, Sjogren’s syndrome, pemphigous vulgaris, bullous pemphigoid, ANCA vasculitis, anti-NDMA (N-methyl D-aspartate) receptors encephalitis, IgA nephropathy, neuromyelitis optica, autoimmune hemolytic anemia, and Buerger's diseases.

^δ^Other immunosuppressive conditions included exogenous steroid use, Cushing’s syndrome, and uncontrolled diabetes.
